# OsRLCK 57, OsRLCK107 and OsRLCK118 Positively Regulate Chitin- and PGN-Induced Immunity in Rice

**DOI:** 10.1186/s12284-017-0145-6

**Published:** 2017-02-21

**Authors:** Zhangqun Li, Ying Ao, Dongru Feng, Jun Liu, Jinfa Wang, Hong-Bin Wang, Bing Liu

**Affiliations:** State Key Laboratory of Biocontrol and Collaborative Innovation Center of Genetics and Development, Guangdong Provincial Key Laboratory of Plant Resources, School of Life Sciences, Sun Yat-sen University, 510275 Guangzhou, People’s Republic of China

**Keywords:** Receptor-Like Cytoplasmic Kinases, Microbe-Associated Molecular Pattern, Pattern Triggered Immunity, Rice

## Abstract

**Background:**

The receptor-like cytoplasmic kinase, OsRLCK176 has been reported to participate in both chitin- and PGN-induced immunity in rice. Here, we further researched the function of the homologous proteins, OsRLCK57, OsRLCK107 and OsRLCK118, in chitin- and PGN immunity in rice.

**Findings:**

Silencing of *OsRLCK57,OsRLCK107* and *OsRLCK118* suppressed chitin- and PGN-induced immunity responses, including reactive oxygen species generation, defense gene expression. Furthermore, OsRLCK107 could interact with OsCERK1 in a MAMP induced way, which suggested a possible physiological relevance of OsRLCKs107 to OsCERK1 pathway.

**Conclusions:**

OsRLCK57, OsRLCK107 and OsRLCK118, positively regulate chitin- and PGN- induced responses in rice, similar to that observed in OsRLCK176.

**Electronic supplementary material:**

The online version of this article (doi:10.1186/s12284-017-0145-6) contains supplementary material, which is available to authorized users.

## Findings

**Fig. 1:**
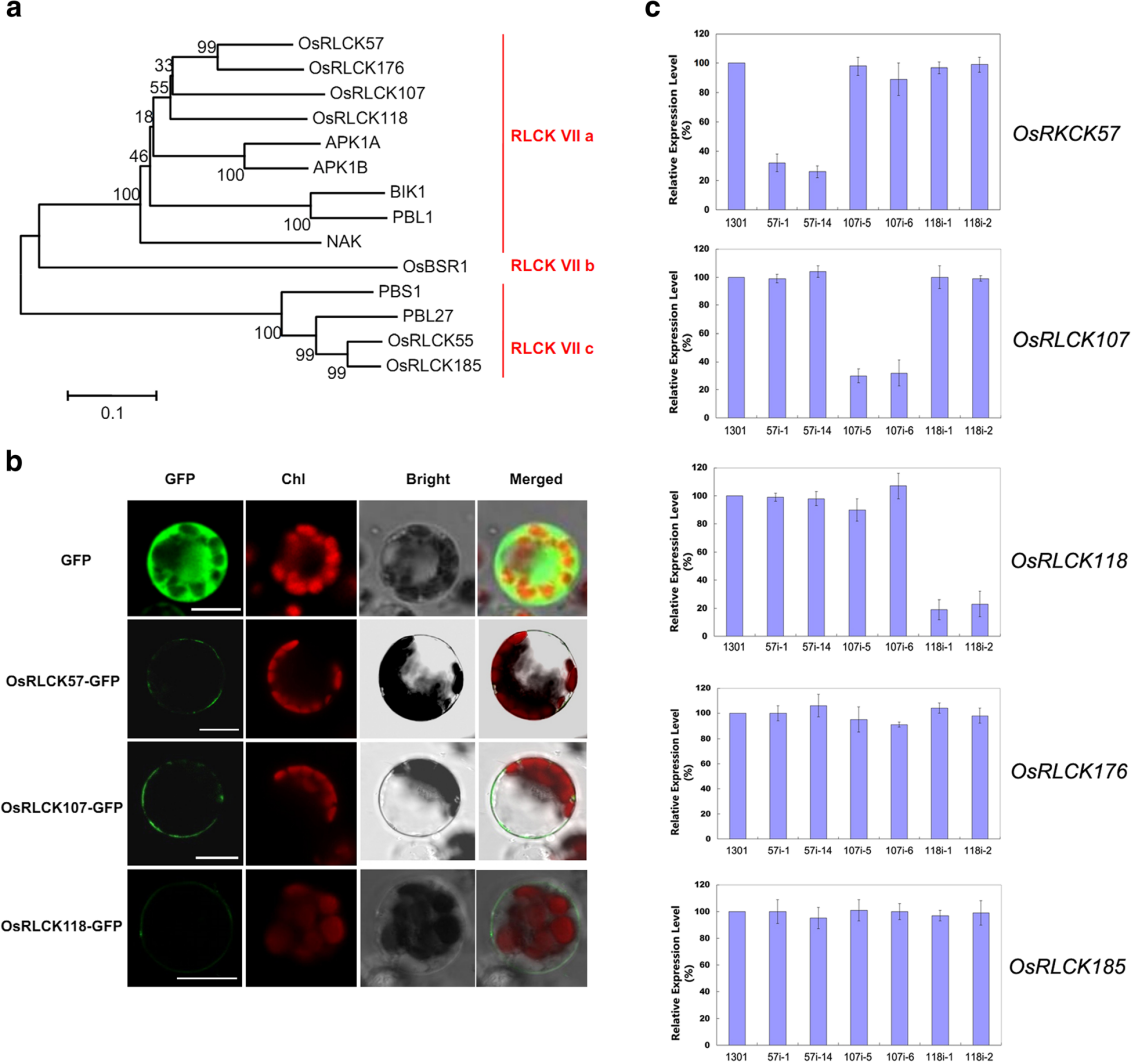
Characterization of homologous OsRLCK57,OsRLCK107 and OsRLCK118. **a** OsRLCK57, OsRLCK107, OsRLCK118 is highly homologous with OsRLCK176 and BIK1. The phylogenetic tree was generated by MEGA4. Full length amino acid sequences were selected to generate the bootstrap neighbor-joining phylogenetic tree. Bootstrap probabilities were obtained from 1000 replicates. Scale bar is indicated. **b** OsRLCK57, OsRLCK107 and OsRLCK118 is plasma membrane localized protein. Subcellular localization of OsRLCK57, OsRLCK107 and OsRLCK118 was visualized using confocal microscope after transiently transformed OsRLCK57-GFP, OsRLCK107-GFP and OsRLCK118-GFP into rice protoplasts. Chl indicated the chlorophyll autofluorescence. Three biological replicates were performed, similar results were obtained. Bar = 10 μm. **c** Expression identification of *RLCKs* in different RNAi lines. The relative expression levels *OsRLCK57*, *OsRLCK107* and *OsRLCK118* were examined by quantitative RT-PCR in *OsRLCK57*, *OsRLCK107* and *OsRLCK118* RNAi lines, respectively. The expression of *OsRLCK176* and *OsRLCK185* was also examined in these RNAi lines to exclude its effects. Empty vector (1301) transgenic callus cells were used as control. 57i-1 and 57i-14: RNAi lines of *OsRLCK57.* 107i-5 and 107i-6: RNAi lines of *OsRLCK107*. 118i-1 and 118i-2: RNAi lines of *OsRLCK118*. The data represents the mean ± standard deviation (SD) from three biological replicates

Plants are constantly challenged by a broad diversity of microbes in the environment. As sessile organisms without specialized immune cells, plants have evolved an elegantly sophisticated defense system, known as “innate immunity”, to identify and eliminate the effects of detrimental invaders (Ausubel 2005). Microbe-associated molecular pattern (MAMP) triggered immunity (PTI) functions at the early stage of innate immunity (Schwessinger and Ronald 2012). The PTI response includes perceptions of MAMPs by pattern recognition receptors (PRRs) at the cell surface, followed by subsequent signal transduction into the cytoplasm to trigger immunity. At the cell surface, lysin motif (LysM) containing receptor-like kinases (RLKs), or non-kinase receptor proteins (RLPs), are the major receptors of oligosaccharidic MAMPs, such as chitin or peptidoglycan (PGN); examples are CEBiP for chitin, LYP4 and LYP6 for chitin and PGN in rice (Kaku et al. 2006; Liu et al. 2012), CERK1, LYK4 and LYK5 for chitin, and LYM1 and LYM3 for PGN in Arabidopsis (Cao et al. 2014; Miya et al. 2007; Wan et al. 2012; Willmann et al. 2011). After perception of MAMPs, a ligand-inducible complex can form to trigger downstream PTI responses. With regard to the signaling components immediately downstream of PRRs, receptor-like cytoplasmic kinases (RLCKs) have been suggested to play critical roles in delivering the immune responses (Macho and Zipfel 2014). Several RLCKs, especially those in subfamily VII, have been reported to participate in PTI in plants. In rice, examples are OsRLCK185 in chitin-induced immunity and OsRLCK176 in both PGN- and chitin-induced immunity in rice (Ao et al. 2014; Yamaguchi et al. 2013). In Arabidopsis, besides the well-characterized roles of BIK1 and PBL1 in flg22-induced immunity, the ortholog of OsRLCK185, PBL27, also functions as a downstream component of CERK1 and contributes to chitin-induced immunity (Lu et al. 2010; Shinya et al. 2014). Recently, Zhou et al. reported that OsRLCK57, OsRLCK107, OsRLCK118 and OsRLCK176 are involved in XA21-mediated immunity and silencing of these genes also compromised the up-regulation of two *PR* genes after chitin treatment (Zhou et al., 2016). Since OsRLCK57, OsRLCK107, OsRLCK118 and OsRLCK176 all belong to RLCK group VIIa (Fig. 1a, Additional file 1: Figure S1) and we previously reported that OsRLCK176 is involved in the chitin and PGN signal response in rice (Ao et al. 2014), we were interested to determine the function of these homologous proteins, OsRLCK57, OsRLCK107 and OsRLCK118, in chitin and PGN immunity in this study.

**Fig. 2:**
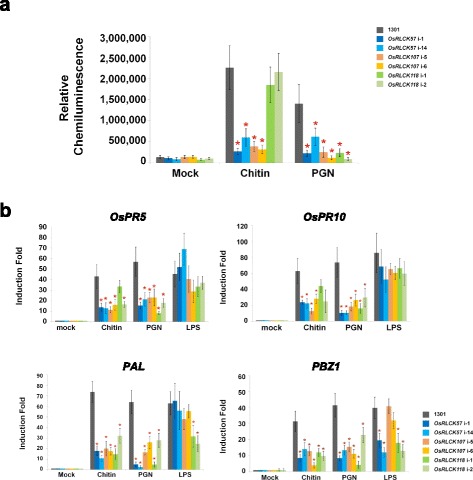
OsRLCK57, OsRLCK107 and OsRLCK118 are involved in chitin- and PGN-triggered immunity in rice. **a** RNAi silencing of *OsRLCKs* compromise the burst of ROS induced by chitin and PGN. *OsRLCK57*-RNAi, *OsRLCK107*-RNAi, *OsRLCK118*-RNAi or empty vector (1301) transgenic callus cells were treated with 100 μg/mL chitin, PGN or sterile water (for mock) for 120 min in dark before assayed. The data represents the mean ± standard deviation (SD) from three biological replicates. Statistically significant differences for *OsRLCK*-RNAi compared with the 1301 were calculated using Student’s *t*-test and indicated by asterisks (*P < 0.05). **b** RNAi silencing of *OsRLCK*s compromise the induction of PR genes by chitin and PGN. *OsPR5*, *OsPR10*, *PAL* and *PBZ1* were determined by quantitative RT-PCR after 6 h treatments with 100 μg/mL chitin, PGN, LPS or sterile water (for mock) in dark. Empty vector (1301) transgenic callus cells were used as control. The data represents the mean ± standard deviation (SD) from three biological replicates. Statistically significant differences for *OsRLCK-*RNAi compared with the 1301 were calculated using Student’s *t*-test and indicated by asterisks (*P < 0.05)

Firstly, we identified the cell surface localization of OsRLCK57, OsRLCK107 and OsRLCK118 by the green fluorescent protein (GFP) fused OsRLCKs in rice protoplasts (Fig. 1b, Additional file 2: Material and Methods). Furthermore, the RNA interference (RNAi) lines with reduced expression of *OsRLCK57*, *OsRLCK107* or *OsRLCK118*, respectively, but not other homologous OsRLCKs, were generated to probe the biological function of these *OsRLCKs* in chitin and PGN signal transduction (Fig. 1c, Additional file 2: Materials and Methods). Two representative RNAi lines were used for each gene in the subsequent analysis and the empty vector (1301) transformed callus was used as a control line. We investigated the reactive oxygen species (ROS) burst, one of the well-established early defense responses in plant immunity, after both chitin and PGN elicitation. We found that production of ROS was significantly induced by chitin in control line 1301, while ROS production was remarkably reduced in these OsRLCKs RNAi lines except the *OsRLCK118* lines (Fig. 2a). The ROS production was reduced to 12–27% in the *OsRLCK57* RNAi lines and to 14–17% in the *OsRLCK107* lines, respectively, compared with control line (Fig. 2a). In PGN treatment assay, all of the ROS production was reduced in different *OsRLCK* RNAi lines, compared with control line 1301 (Fig. 2a). Inhibition of the ROS burst indicated that OsRLCK57, OsRLCK107 and OsRLCK118 could be involved in chitin- and/or PGN-induced immunity in rice, similar to OsRLCK176 and OsRLCK185 (Ao et al. 2014; Yamaguchi et al. 2013). Moreover, we investigated the activation of defense genes, a common indicator of the resistance response in plant immunity, in the RNAi lines. Four representative defense genes, *OsPR5, OsPR10, PAL* and *PBZ1*, which were previously examined in the *OsRLCK176*-RNAi lines and/or the *OsRLCK185*-RNAi lines, were assayed in this research (Ao et al. 2014; Yamaguchi et al. 2013). After chitin or PGN treatment, all of the four selected *PR* genes were induced significantly in the control line, while expression of these genes was significantly suppressed in the *OsRLCK*-RNAi lines (Fig. 2b). As control, after another MAMP, LPS (lipopolysaccharide) treatment, the defense genes activation was only markedly suppressed in *OsRLCK118*-RNAi lines (for *PAL* gene) and *OsRLCK57* and *OsRLCK118*-RNAi lines (for *PBZ1* gene) (Fig. 2b). Both the compromised burst of ROS and the activation of defense genes indicate that *OsRLCK57, OsRLCK107, OsRLCK118* and *OsRLCK107* play important roles in chitin- and PGN-induced defense responses in rice, just as *OsRLCK185* and *OsRLCK176* do.

**Fig. 3:**
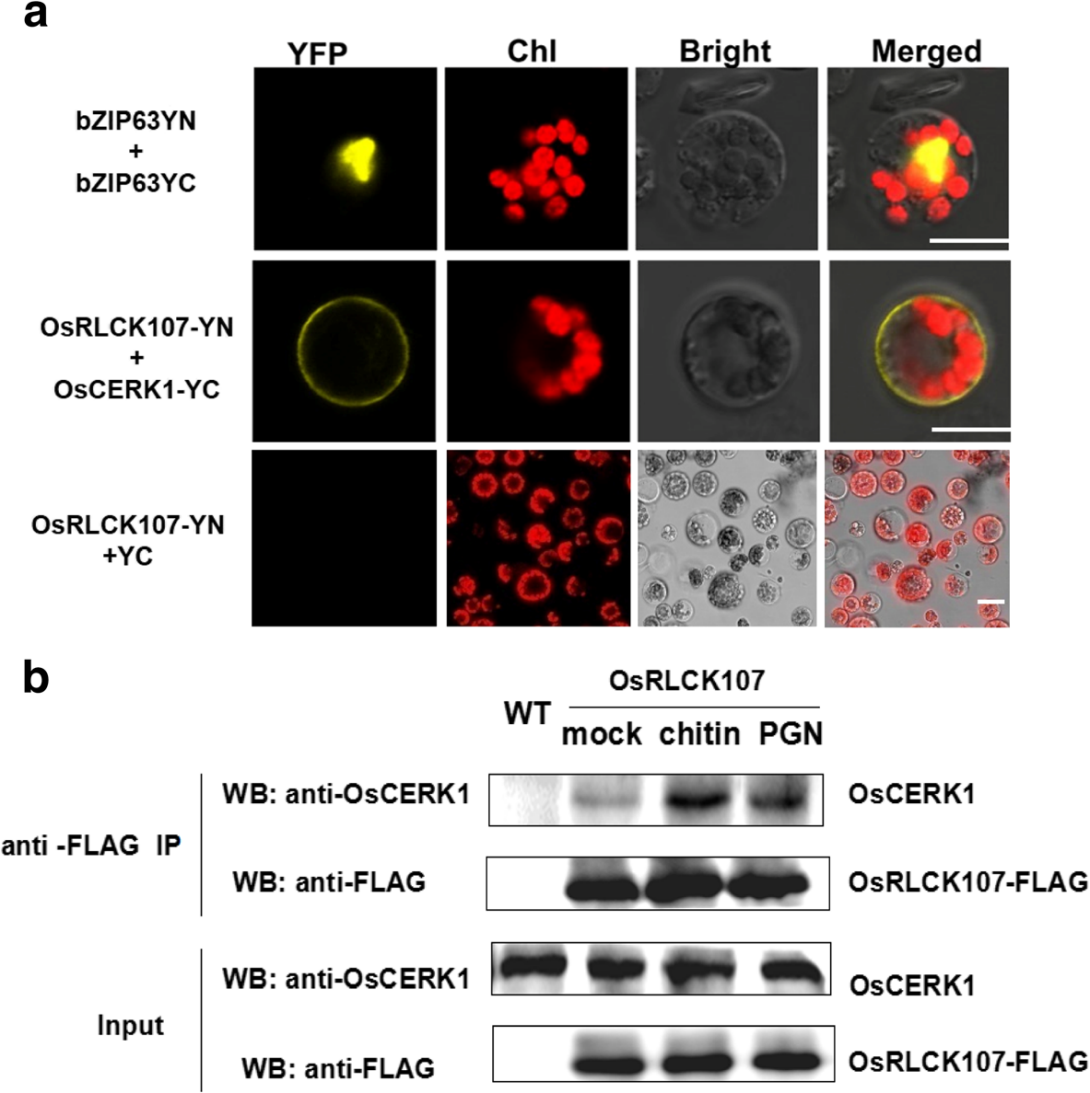
OsRLCK107 interacts with OsCERK1. **a** OsRLCK107 interacts with OsCERK1 by BiFC. OsRLCKs-YN and OsCERK1-YC were co-expressed in rice protoplasts, the reconstituted YFP fluorescence was visualized using confocal microscope. Chl indicated the chlorophyll autofluorescence. Positive control: co-expression of bZIP63-YN and bZIP63-YC, negative control: co-expression of OsRLCKs-YN and blank-YC. Bar = 10 μm. **b** The interaction between OsRLCK107 with OsCERK1 is regulated by chitin and PGN. Protoplasts expressing OsRLCK107 with a C-terminal 3 × FLAG epitope tag were treated with 150 μg/mL chitin、PGN or sterile water (for mock) for 10 min. After treatment, protein was prepared for immunoprecipitation with anti-FLAG GEL (Sigma). The presence of OsCERK1 in the complex was analyzed by OsCERK1 antibody. Protein exacted from wide type (WT) protoplasts was used as negative control. Three biological replicates were performed and similar results were obtained

In chitin- and PGN-trigged innate immunity in rice, OsCERK1 integrates extracellular chitin and PGN signals from different PRRs and delivers the immune signals to the cytoplasm by interacting with its cytoplasmic targets (Akamatsu et al. 2013; Ao et al. 2014; Shimizu et al. 2010; Yamaguchi et al. 2013). We were interested to determine whether OsRLCK57, OsRLCK107 and OsRLCK118 were correlated with OsCERK1. The Bimolecular fluorescence complementation (BiFC) assay indicated the interaction between OsRLCK107 with OsCERK1 (Fig. 3a). The interaction of OsRLCK107 with OsCERK1 was further confirmed by co-immunoprecipitation (coIP) analysis in rice protoplasts (Fig. 3b). However the interaction was not observed with OsRLCK55 and 118, possible due to the low expression of these proteins in our system. Interestingly, in the reported regulation mechanism of PRR-RLCKs in rice, OsRLCK176 and OsRLCK185 associate with OsCERK1 in the absence of MAMPs, and they then disassociate from OsCERK1 in the presence of MAMPs (Ao et al. 2014; Yamaguchi et al. 2013). Whereas the interaction between OsRLCK107 and OsCERK1 was enhanced in the presence of chitin or PGN (Fig. 3b), which distinguishes it from the reported actions of OsRLCK176 and OsRLCK185. The different mechanism of OsRLCKs in chitin and PGN signaling may indicate the complexity of the intracellular signaling network downstream of plasma membrane receptors.

PTI requires cytoplasmic members to link PRR activation with downstream signaling and RLCKs have emerged as key roles in the mechanism. In this research, we identified three members of RLCK group VIIa, OsRLCK57, OsRLCK107 and OsRLCK118, play roles in chitin and PGN immunity in rice. These results presented here will provide novel insight into the downstream signaling regulation of the chitin and PGN immune responses in rice.

## Additional Files

Additional file 1: Figure S1. Aligments of full length amino acid sequences of OsRLCK57, OsRLCK107, OsRLCK118 and other RLCKs. **Table S1.** Primers used in this study. (DOC 6724 kb)
Additional file 2: Materials and Methods. (DOC 59 kb)

## Abbreviations

MAMP: Microbe-Associated Molecular Pattern
PGN: Peptidoglycan
PRR: Pattern Recognition Receptor
PTI: Pattern Triggered Immunity
RLCK: Receptor-Like Cytoplasmic Kinase
