# Supplementary material for: OsRLCK 57, OsRLCK107 and OsRLCK118 Positively Regulate Chitin- and PGN-Induced Immunity in Rice
Source: Rice (N Y). 2017 Feb 21;10:6. doi: 10.1186/s12284-017-0145-6 (PMC5318303; doi:10.1186/s12284-017-0145-6)
Supplement: Additional file 1: Figure S1. — Aligments of full length amino acid sequences of OsRLCK57, OsRLCK107, OsRLCK118 and other RLCKs. Table S1. Primers used in this study. (DOC 6724 kb) [file 12284_2017_145_MOESM1_ESM.doc]

**Additional file 1: Figure S1**


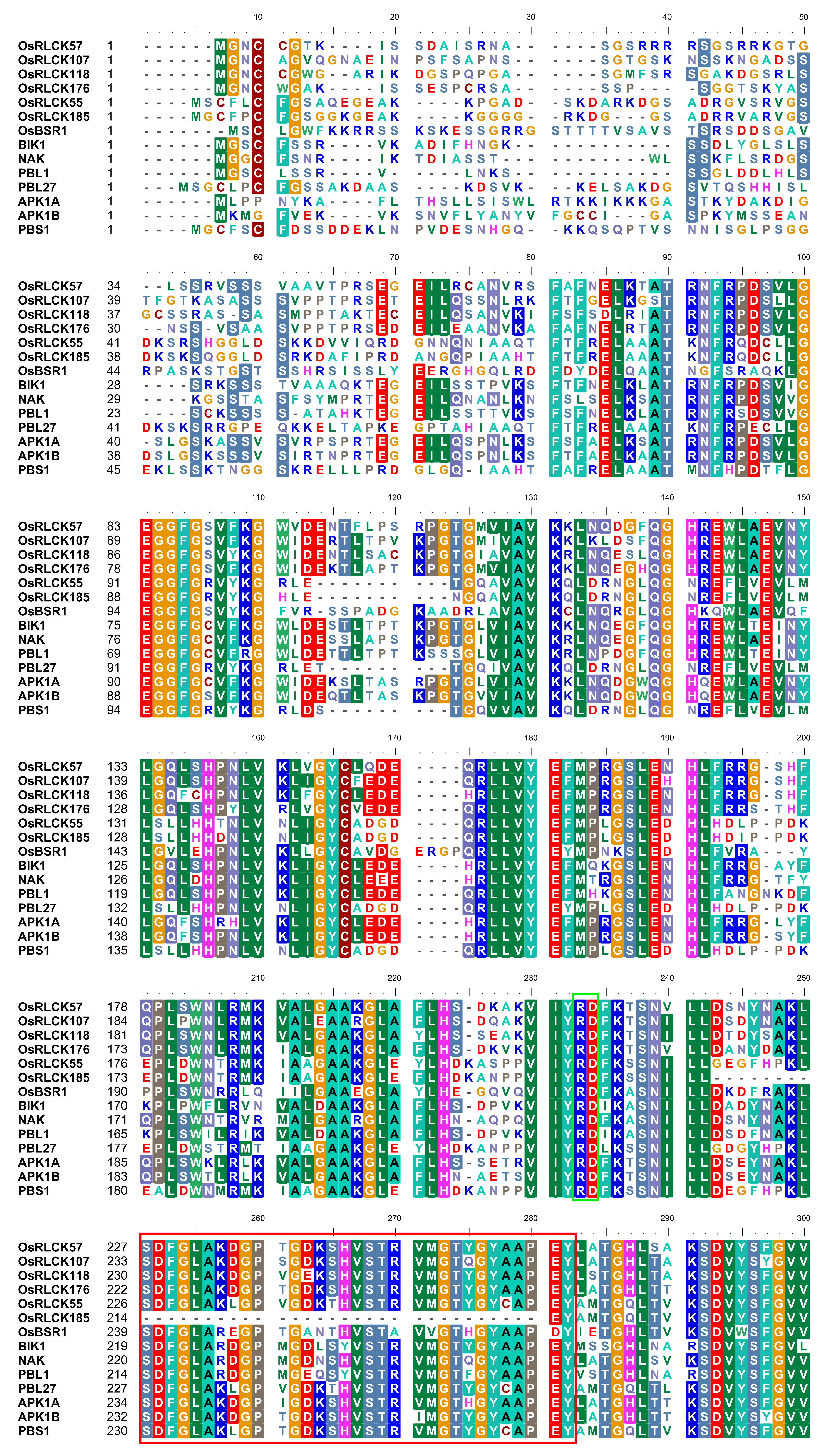


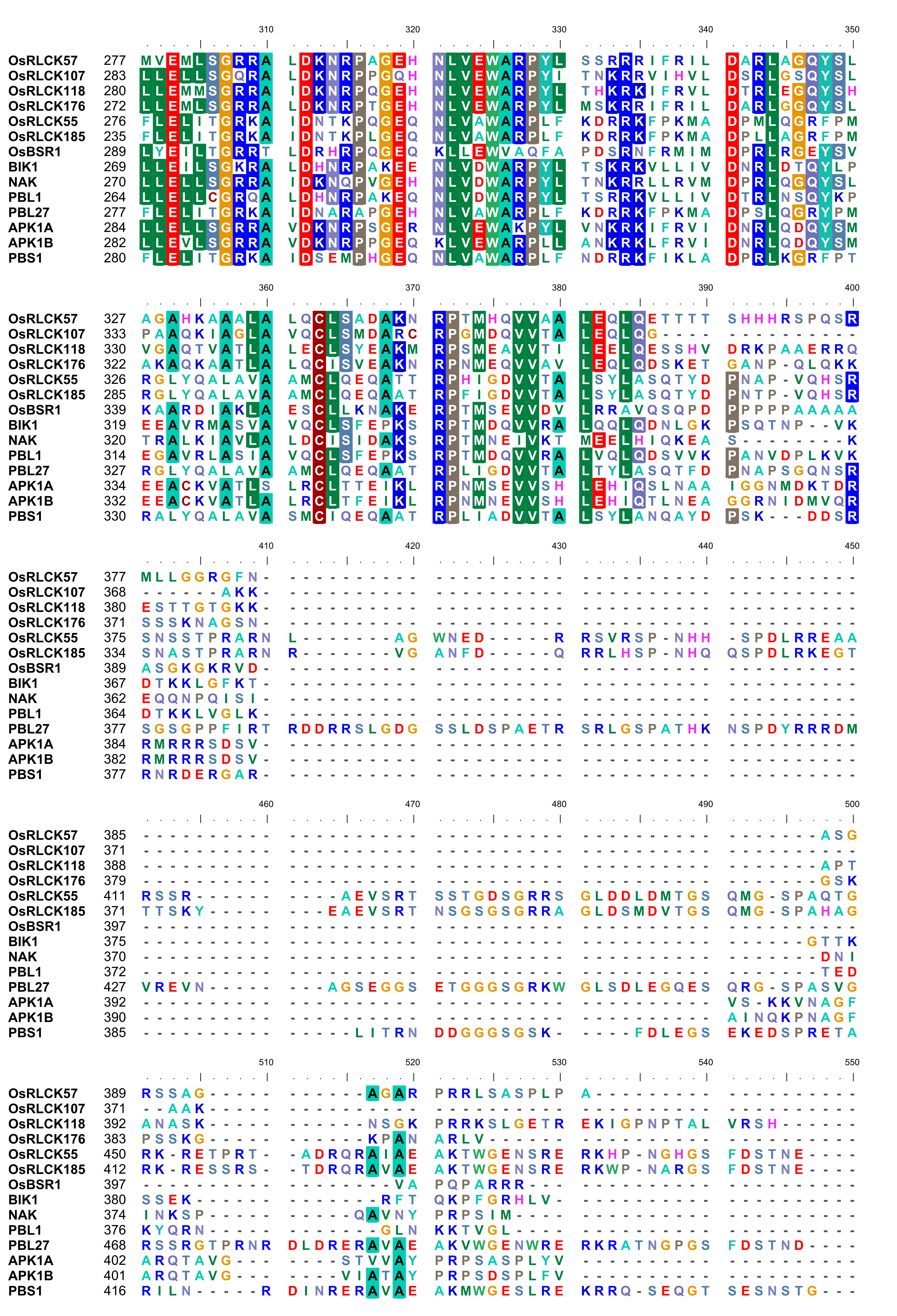


**Additional Figure 1. Aligments of full length amino acid sequences of OsRLCK57, OsRLCK107, OsRLCK118 and other RLCKs.**

Aligments were conducted by Bioedit. Threshhold for shading was 50%. The black box indicates RD domain and the red box indicates activation domain of kinases.

**Additional file 1: Table S**1 Primers used in this study

| OsRLCK57 RNAi-sence-F | 5'-CTGCAGATCCAGGACTTTGATG-3' |
| --- | --- |
| OsRLCK57 RNAi-sence-R | 5'-AAGCTTCCGTCTTGAGCTCGTT-3' |
| OsRLCK57 RNAi-antisence-F | 5'-ACTAGTATCCAGGACTTTGATGATC-3' |
| OsRLCK57 RNAi-antisence-R | 5'-GTCGACCCGTCTTGAGCTCGT-3' |
| OsRLCK107 RNAi-sence-F | 5'-CCATGGGAAGGGGACGCTG-3' |
| OsRLCK107 RNAi-sence-R | 5'-AAGCTTAGCCCTTGAGCTCGC-3' |
| OsRLCK107 RNAi-antisence-F | 5'-ACTAGTGAAGGGGACGCTGG-3' |
| OsRLCK107 RNAi-antisence-R | 5'-GTCGACAGCCCTTGAGCTCG-3' |
| OsRLCK118 RNAi-sence-F | 5'-CCATGGAGCAGCCAGCAG-3' |
| OsRLCK118 RNAi-sence-R | 5'-AAGCTTCTATCCTGAGATCACTAAAAC-3' |
| OsRLCK118 RNAi-antisence-F | 5'-ACTAGTAGCAGCCAGCAGCAG-3' |
| OsRLCK118 RNAi-antisence-R | 5'-GTCGACCTATCCTGAGATCACT-3' |
| OsRLCK57-GFP/YN-F | 5'-TCTAGAATGGGCAACTGCTGCGGAACC-3' |
| OsRLCK57-GFP/YN-R | 5'-TATGGTACCGGCAGGCAGAGGCGAC-3' |
| OsRLCK107-GFP/YN/FLAG-F | 5'-TCTAGAATGGGGAACTGCGCCG-3' |
| OsRLCK107-GFP/YN/FLAG-R | 5'-GGTACCCTTGGCTGCTTTCTTTGCA-3' |
| OsRLCK118-GFP/YN-F | 5'-TCTAGAATGGGGAATTGCTGTGGGTGGGGC-3' |
| OsRLCK118-GFP/YN-R | 5'-GGTACCATGGGAGCGGACCAATGCTGTT-3' |
| OsRLCK176-FLAG-F | 5'-TCTAGAATGGGGAATTGCTGGGGCGC-3' |
| OsRLCK176-FLAG-R | 5'-GGTACCAACCAGCCTCGCATTTGCG-3' |
| OsCERK1-YC-F | 5'-TCTAGA ATGGAAGCTTCCACCTC-3' |
| OsCERK1-YC-R | 5'-GGTACCTCTCCCGGACATTAGGTT-3' |
| qOsRLCK57-F | 5'-TCAGGAGACGACGACCACG-3' |
| qOsRLCK57-R | 5'-GAGCACCAGCACCAGCAGA-3' |
| qOsRLCK107-F | 5'-TCCATGTCCTCGACTCACG-3' |
| qOsRLCK107-R | 5'-TGCATCCATTGACAGGCAC-3' |
| qOsRLCK118-F | 5'-GTTGCCACCCTTGCTCT-3' |
| qOsRLCK118-R | 5'-CTGCTGGTTTCCTGTCC-3' |
| qOsRLCK176-F | 5'-GAGCAGGTTGTTGCTGTATT-3' |
| qOsRLCK176-R | 5'-CCCTTCGATGACGGTTTC-3' |
| qOsRLCK185-F | 5'-AGACACCACCGCTGCTTCA-3' |
| qOsRLCK185-R | 5'-TCGCGGTGATGCTATTCGTA-3' |
| actin-F | 5'-AGGCTCCTCTCAACCCCAAG-3' |
| actin-R | 5'-TTTCCTGGTCATAGTCCAGG-3' |
| qOsPR5-F | 5'-CGCTGCCCCGACGCTTAC-3' |
| qOsPR5-R | 5'-ACGACTTGGTAGTTCTGTTGC-3' |
| qOsPR10-F | 5'-CCTCAGCCATGCCATTCAG-3' |
| qOsPR10-R | 5'-CTTGTCCACGTCCAGGAACTC-3' |
| qPBZ1-F | 5'-GGTGTGGGAAGCACATACAA-3' |
| qPBZ1-R | 5'-GTCTCCGTCGAGTGTGACTTG-3' |
| qPAL-F | 5'-TGAATAACAGTGGAGTGTGGAG-3' |
| qPAL-R | 5'- AACCTGCCACTCGTACCAAG-3' |
