# Supplementary material for: OsRLCK 57, OsRLCK107 and OsRLCK118 Positively Regulate Chitin- and PGN-Induced Immunity in Rice
Source: Rice (N Y). 2017 Feb 21;10:6. doi: 10.1186/s12284-017-0145-6 (PMC5318303; doi:10.1186/s12284-017-0145-6)
Supplement: Additional file 2: — Materials and Methods. (DOC 59 kb) [file 12284_2017_145_MOESM2_ESM.doc]

**Additional file 2**

**Material and Methods:**

**Stable transformation**

Callus cells were transformed as previously described with some modifications . Briefly, de-hulled and sterilized rice seeds were cultured on N6D solid medium under continuous light at 32℃ for 8 days to yield calli. Agrobacterium strains EHA105 harboring targeting sequence were cultured on YEP solid medium containing 50 μg /mL kanamycin sulfate and rifampin at 28℃ in the dark. The agrobacterium were scraped from the plates and suspended in AAM-As medium (liquid). Yielded native calli were immersed into the agrobacterium suspension, with gently shaking for 5min. Then calli were transferred onto N6-As medium pre-placed with a sterilized ﬁlter paper which was soaked by AAM-As liquid medium. After 3 day’s co-cultivation at 28℃ in the dark, the calli were washed several times with sterilized water to remove agrobacterium away and removed to N6D solid medium containing 50μg /mL hygromycin and 400μg /mL carbenicillin. A continuous light at 32℃ about 2 weeks is required to yield transformed calli. Calli selected by 50μg /mL hygromycin were cultivated under 25℃ in the dark and transferred into fresh medium every two weeks. The vigorous callus cells were transferred into fresh N6D liquid medium for three days before MAMP treatment.

**Plasmid construction**

Routine molecular cloning techniques were followed to make the constructs. The primers used in this work are listed. For transient expression, target CDS were cloned from rice cDNA, and then built into plant transient expression vector with epitope tag. For stable RNAi constructs, the sense and antisense fragments were cloned from rice DNA, and inserted into the pCAMBIA1301, fragments were separated by an intron and expressed under the control of monocot specific Act1 promoter. After validation, the recombined constructs were transformed into Agrobacterium EHA105 for stable transformation. For GFP fusion constructs, the GFP was fused with C-terminal of OsRLCKs and then driven by a 35S promoter inserted in a PUC18 vector. For BiFC construct, the N-terminal 155 amino acids (YFP^N^) was fused with OsRLCK and the C-terminal 86 amino acids of YFP (YFP^C^) was fused with OsCERK1. The fused constructs were then driven by the 35S promoter inserted in a PUC18 vector.

**MAMP preparation**

Chitin used was purchased from Sigma (Cat # C9752). Lipopolysaccharide (LPS) was purchased from Sigma (Cat # L2630). PGN used were purified from *Xanthomonas oryzae pv. oryzicola* as previously described .

**Bioinformatic Analysis**

The full length amino acid alignments of RLCKs were performed by BioEdit. The phylogenetic analysis of rice and Arabidopsis thaliana RLCKs were performed by MEGA4 after alignment with BioEdit .

**Identification of RNAi lines**

Total RNA was extracted from different rice calli lines selected by 50μg/mL hygromycin. Then gene-specific qPCR was conducted to determine the specificity and efficiency of RNA interference of *OsRLCK57, OsRLCK 107* and *OsRLCK* 118, respectively.

**Analysis of PR genes**

Vigorous callus cells cultivated under 25℃ in continuous dark were transferred into fresh N6D liquid medium for 3 days. Then, callus cells were used for treatment with 100 µg/ml chitin, PGN, LPS or sterilized water (for mock) in fresh N6D liquid medium for 6h in dark. After treatment, total RNA was extracted and gene-specific qPCR was conducted to determine the induction of PR genes.

**Quantitative real-time PCR**

Total RNA was extracted by RNeasy Plant Mini Kit (Omega, USA). The first strands of cDNA were reverse transcribed by PrimeScript RT Reagent Kit (Takara, Japan). Gene-specific qPCR was then conducted with SYBR ® Premix Ex Taq™ (Takara) on RoChe LC480 to analysis mRNA abundance. Rice *Actin1* was used as internal control.

**ROS measurement**

ROS burst was determined by chemiluminescence as previously described . Briefly, vigorous callus cells cultivated under 25℃ in continuous dark were transferred into fresh N6D liquid medium for 3 days. Then, callus cells were retransferred into medium containing 0.5 mM L-012 (Wako, Japan) and 100 µg/ml chitin, PGN or sterilized water (for mock). The chemiluminescence was assayed at 120 min after elicitor treatment with TriStar² LB 942, Berthold at a time set of 5 seconds.

**Transient expression**

Isolation and protoplast-mediated transient expression was performed as previously described . Briefly, 8-10 day-old seedlings grown on 1/2MS, 25℃, 12h light/12h dark were cut into 0.5mm strips and incubated in the enzyme solution (0.6M mannitol, 10mM MES (pH5.7), 0.75% Macerozyme R-10, 1.5% Cellulase RS, 10 mM CaCl_2_ and 0.1% BSA) for 4-5 h in the dark with gentle shaking (60 to 80 rpm). After enzymatic digestion in dark for 4-5h, an equal volume of W5 (2 mM MES (pH 5.7), 5 mM KCl, 154 mM NaCl and 125 mM CaCl_2_) was added, then pellets were filtered through a 40 μm nylon meshes into tubes after vigorous shakes for 10 second. Protoplasts were collected by centrifugation at 1,500 rpm for 3 min. After washing by W5 solution once, protoplasts were then resuspended in MMG solution (4 mM MES (pH 5.7), 0.4 M mannitol, 15 mM MgCl_2_) at a final concentration of 2 × 10^6^cells/mL.

For PEG-mediated transfections, total 100μg plasmid DNA were added into per 1mL protoplasts, then 1100mL freshly prepared PEG solution (0.2 M mannitol, 0.1 M CaCl _2_ and 40% (W/V) PEG 4000) was added, and mixture by gently inverting the tube. After 15min incubation of the resulting solution in dark, 4400μ L W5 solution were added slowly to stop the transfection. After centrifugation at 1,500 rpm for 3 min, the protoplasts were resuspended gently in WI solution (0.5 M mannitol, 20 mM KCl and 4 mM MES (pH 5.7)). Finally, the protoplasts were transferred into 24-well plates or plates, and cultured in dark at 25℃ for 12 h.

For GFP/BiFC, 100 to 200μ L transfected protoplasts were enough for observation by the confocal laser scanning microscope (Leica TCS 5 SP5 AOBS). For coIP assay, much more transfected protoplasts were needed.

**Coimmunoprecipitation assay**

The assay was performed according to previously reported . Briefly, the coding sequences of target were amplified by PCR from rice cDNA and inserted into plant transient expression vector with 3×FLAG epitope tag at C-terminal, resperctively. The recombined vector was transiently-expressed in protoplasts as above. For eliciting, 150 µg/ml chitin, PGN or sterilized water (for mock) were added, and inclubated for 15min with gentle shaking in dark at 25℃. Total protein was extracted by vortex-shocking from transfected protoplasts with extraction buffer (50 mM HEPES, 150 mM KCl, 1 mM DTT, 1 mM EDTA, 0.5% TritonX-100, and protease inhibitor cocktail (Roche, Switzerland) pH 7.5). After centrifugation, protein was incubated with agarose-conjugated anti-FLAG antibody (Sigma, A2220) for 4 h with gentle shaking. The beads were collected and washed four times. Interaction proteins were examined by WB.

**OsCERK1 polyclonal antibody**

The OsCERK1 antibody used in this study was described as previous .

**References**

Ao, Y., Li, Z., Feng, D., Xiong, F., Liu, J., Li, J.F., Wang, M., Wang, J., Liu, B., and Wang, H.B. (2014). OsCERK1 and OsRLCK176 play important roles in peptidoglycan and chitin signaling in rice innate immunity. Plant J. **80**:1072-1084.

Liu, B., Li, J.F., Ao, Y., Qu, J., Li, Z., Su, J., Zhang, Y., Liu, J., Feng, D., Qi, K., et al. (2012). Lysin motif-containing proteins LYP4 and LYP6 play dual roles in peptidoglycan and chitin perception in rice innate immunity. Plant Cell **24**:3406-3419.

Toki, S., Hara, N., Ono, K., Onodera, H., Tagiri, A., Oka, S., and Tanaka, H. (2006). Early infection of scutellum tissue with Agrobacterium allows high-speed transformation of rice. Plant J. **47**:969-976.

Zhang, Y., Su, J., Duan, S., Ao, Y., Dai, J., Liu, J., Wang, P., Li, Y., Liu, B., Feng, D., et al. (2011). A highly efficient rice green tissue protoplast system for transient gene expression and studying light/chloroplast-related processes. Plant methods **7**:30.
